# Migration, Partner Selection, and Fertility in Germany: How Many Children are Born in Mixed Unions?

**DOI:** 10.1007/s10680-024-09710-w

**Published:** 2024-06-28

**Authors:** Annegret Gawron, Nadja Milewski

**Affiliations:** 1 Institute of Sociology and Demography, University of Rostock, Ulmenstraße 69, 18057 Rostock, Germany; 2 Federal Institute for Population Research, Wiesbaden, Germany

**Keywords:** Exogamy, Migration, Fertility, Germany

## Abstract

For the German context, we investigate whether the number of children ever born differs between mixed unions (exogamous unions between natives and migrants or migrant descendants) and endogamous unions (unions among co-ethnics). Our theoretical considerations are derived from assimilation theories, which view exogamous unions as indicators of assimilation processes, and the framework on migrant fertility. The migrant (or descendant) partner in an exogamous union may adapt to the majority group, both partners may adapt to each other, or both partners may constitute a selected group in their fertility preferences. However, due to the higher likelihood of conflicts within the partnership and of separation, exogamy may disrupt family formation processes and depress couples’ fertility. Drawing on data from the GSOEP (1984–2020), we estimate generalized Poisson regressions. The results reveal that the number of children ever born is higher in exogamous unions than in endogamous native couples. This general pattern largely persists across migrant generations and regions of origin, but we identify gender differences. While fertility in exogamous unions of native women/migrant (descendant) men is not statistically different from fertility in native/native couples, unions of migrant (descendant) women/native men have more children, especially when controlling for socio-demographic confounders. Our results demonstrate that in the German context, exogamy does not lead to fertility disruptions, and is not straightforwardly associated with assimilation to the fertility of the majority group. Instead, differences in gendered partner choice patterns and life-course transitions may influence the number of children exogamous couples have.

**Supplementary Information:**

The online version contains supplementary material available at 10.1007/s10680-024-09710-w.

## Introduction

Over the past two decades, numerous studies have investigated fertility among migrant populations (Kulu et al., 2019). Particularly in countries where fertility has fallen below the replacement level (Morgan, 2003), there is substantial research on the fertility of migrant populations. As migrant women (especially from high-fertility countries) living in low-fertility countries have, on average, more children than native women (Milewski & Adserà, 2023), this research interest correlates with the expectation that fertility in migrant populations may increase fertility in low-fertility countries, and that demographic behaviour is an indicator of assimilation processes (Billari, 2008; Goldstein et al., 2009). However, previous research on migrant fertility largely overlooked the fact that fertility usually occurs within partnerships (Bauer & Kneip, 2013, 2014). Increasing international migration and mobility and globalisation processes (Castles et al., 2013) have made the partner market more diverse, and the numbers of (any) social boundary-crossing couples have risen in both majority and (migrant) minority groups (Braack & Milewski, 2020; Lanzieri, 2012). Hence, migrants, migrant descendants, and natives may choose a partner of their own ethnic group (i.e., endogamy) or a partner of a different group (i.e., exogamy), which can have implications for their fertility behaviour. To investigate the association between migration and fertility on the couple level, we pose the following research question: *How many children are born in exogamous unions versus in endogamous unions among native non-migrants?*

By addressing this research question, this article combines two classical indicators of migrant assimilation. The fertility behaviour of migrants and their descendants is seen as an indicator of their cultural and socio-economic adaptation to the majority group (Coleman, 1994). Additionally, exogamous unions signal the crossing of social group boundaries (Alba & Nee, 2003; Carol & Leszczensky, 2019; Qian & Lichter, 2007), and their offspring may show greater “mixedness”, or diversity. Fertility in exogamous pairings blurs ethnic boundaries, and, as ethnic minority status and social status are highly correlated, this boundary blurring may contribute to the transformation of social structures within destination societies (Alba, 2022). However, whether exogamy contributes to the growth of a multi-ethnic population is unclear. Theoretical considerations regarding this issue are not conclusive. On the one hand, exogamy indicates assimilation, and the fertility patterns of exogamous couples may be similar to those of natives. On the other, fertility may be disrupted in exogamous unions because the partners’ values and norms regarding childbearing are dissimilar (Huinink & Konietzka, 2007). Compared to partners in endogamous unions, partners in exogamous unions tend to differ more in their family backgrounds and values, and in other individual traits, like age and education (Elwert, 2020; González-Ferrer et al., 2018). Moreover, even when exogamous partners (i.e., individuals in exogamous unions) have similar fertility preferences, the realisation of their preferences may be hampered, especially given that exogamy is associated with higher union dissolution risks (Choi & Goldberg, 2021; Milewski & Kulu, 2014).

There is limited empirical research on the association between exogamy and fertility. Several previous investigations focused on fertility among interracial couples in the US (Choi & Goldberg, 2018, 2020; Fu, 2008; Qian & Lichter, 2021). Only a few existing studies in Europe have explicitly focused on the impact of exogamy on fertility among migrants and their descendants. Pereiro et al. (2023) examined the fertility intentions of female migrants in exogamous unions in Italy. Elwert (2023) estimated third-birth risks in exogamous unions among female and male migrants in Sweden. Van Landschoot et al., (2017, 2018) analysed the birth transition rates of female migrant descendants with native partners in Belgium. For Germany, Glowsky (2015) investigated the number of children born to male natives with female migrant partners from Poland, Thailand, and Russia. Thus, the question of how fertility intentions and the tempo and the quantum of selected birth transitions translate into the final number of children couples have remains open. As these ideational and behavioural fertility measures may produce different patterns of group differences (e.g., Mussino et al., 2021), investigations of the number of children ever born to couples are needed to understand the long-term impact of mixed unions on demographic developments. Additionally, the current lack of studies on fertility among native women in unions with migrant (descendant) partners mirrors the blind spot of research on the implications of exogamy for the respective majority groups (i.e., natives) in Europe (Braack & Milewski, 2019) and the marginalised position of migrant (descendant) men in fertility studies (Milewski & Baykara-Krumme, 2023).

Our study contributes to the literature in several ways. First, we add to the literature on partnership heterogeneity and fertility by investigating exogamy. Second, we provide insight into the consequences of exogamy across the life course from the perspective of “linked lives” (Elder, 1994) by analysing couples’ fertility. Third, whereas previous research on fertility by union type focused on indicators like fertility intentions or single birth transition rates, we examine couples’ number of children ever born. Fourth, we also analyse exogamous unions between male migrant (descendant)s and female natives (i.e., native non-migrants). Our analyses focus on Germany, which has had low fertility since the 1970s, and has been one of the main destination countries for immigrants for about seven decades. We use generalized Poisson regressions on GSOEP data (1984–2020) to investigate couples’ number of children ever born by distinguishing between endogamous and exogamous (marital and non-marital) unions of natives and (first-, 1.5-, and second-generation) migrants. The analysed unions are based on women’s first and second unions during their reproductive years.

## Theoretical Background

Typical explanations for migrant fertility focus on the roles of socialisation, selection, disruption, and adaptation (e.g., Adsera & Ferrer, 2015; Kulu et al., 2019). The fertility of migrants and their descendants may, for example, be linked to norms, values, and preferences they acquired through socialisation in their country of origin’s majority culture or in a minority group *subculture*. It could also stem from *selection*, e.g., in terms of family size preferences. Moreover, the migration process may be associated with *disruptive* factors (e.g., stress during migration, discrimination experiences) that lead to lower fertility. Migrants and their descendants may also *adapt* their fertility preferences to the economic, social, and cultural conditions in the destination country. The adaptation perspective views the fertility behaviour of migrants and their descendants as an indicator of their cultural and structural assimilation to the destination country (Milewski & Mussino, 2018).

**Fig. 1:**
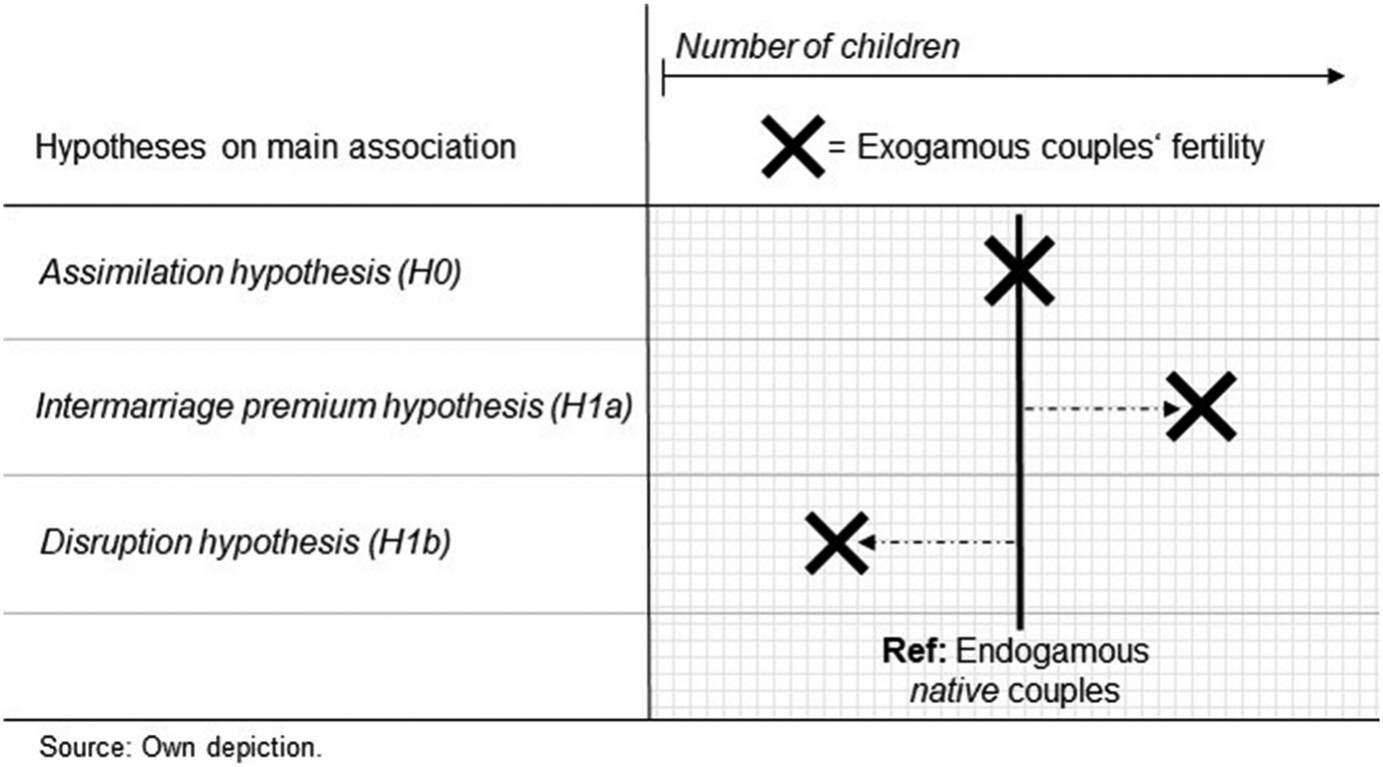
Graphic depiction of the three main hypotheses

Rather than focusing solely on the individual-level fertility of migrants and their descendants, we analyse the implications of exogamous unions for fertility. The few previous studies on the association between exogamy and fertility provided mixed evidence. Some found that fertility in exogamous unions is higher than that in endogamous unions of the majority group (Choi & Goldberg, 2020; Elwert, 2023), whereas others found that fertility in exogamous unions is lower than (Fu, 2008; Glowsky, 2015) or similar to that in endogamous unions of the majority group (Choi & Goldberg, 2018). In the following sections, we take the different empirical findings as points of departure for our theoretical considerations, which build upon the terminologies of the classical hypotheses on migrant fertility, but expand the potential impact of migrant status on fertility on the couple level using literature on migrant assimilation and exogamy. Based on these considerations, we derive hypotheses that specify the position of fertility in exogamous unions relative to that in endogamous native unions (see Fig. 1). We conclude our deliberations on fertility patterns among exogamous couples by summarising the role of selection in these unions. Lastly, we look at within-migrant variation (generation and origin).

### Adaptation as a One-Way Process

We start with findings indicating that exogamous couples’ fertility differs only slightly (or not at all) from that of endogamous unions with majority group members (i.e., natives) (Choi & Goldberg, 2018). The observation that fertility among exogamous couples is similar to that among the majority group may be seen as evidence of *migrant adaptation,* or *assimilation*. Classical assimilation theory assumes that exogamous union formation is the final stage in the assimilation processes of migrant groups (Gordon, 1964). Indeed, cultural (e.g., understanding of the host country’s language, but also its values and norms) and structural assimilation (in socio-economic traits like education and income) may contribute to exogamous union formation among migrants and their descendants (Dribe & Lundh, 2008, 2011; Furtado & Song, 2015; Wiik & Bergsvik, 2023). The link between migrant assimilation and exogamy may have implications for couples’ fertility. For example, fertility behaviour in exogamous unions may be similar to that in endogamous native unions. Similar behaviour might occur when exogamous union formation is linked to migrant (descendant)s consciously departing from their culture of origin (mentioned for women: Collet, 2015), and, thus, potentially from their culture of origin’s fertility norms and values, to more closely align their values with those of the majority group in the destination country. Therefore, having (fertility) preferences that mirror those of the majority group may influence exogamous partner choice among migrants and migrant descendants, and affect the fertility outcomes of their unions.

Higher education is associated with individualistic attitudes and detachment from family and the community of origin (Kalmijn, 1998). Consequently, higher educated migrants and migrant descendants are more likely to adjust their fertility values and norms to those of the majority group. As higher educated migrant (descendant) women and men rather intermarry (Dribe & Lundh, 2008; González-Ferrer, 2006; Wiik & Bergsvik, 2023), exogamous couples’ fertility behaviour may be similar to that of endogamous natives, independent of the makeup of the exogamous union (i.e., whether the woman or the man belongs to a migrant group).

The higher education levels among migrants and migrant descendants in exogamous unions help to explain why they have more favourable labour market positions than their counterparts in endogamous unions (denoted as “intermarriage premium”) (Furtado & Song, 2015; Wiik & Bergsvik, 2023). Given the intermarriage premium, opportunity costs should affect the realisation of fertility preferences among exogamous unions in a manner similar to that among endogamous natives. Note, however, that exogamy may also facilitate migrant (descendant)s’ assimilation to the majority group (Rodríguez-García, 2015). Having ties to natives can also improve exogamous migrant (descendant)s’ cultural adaptation (Rodríguez-García et al., 2015), labour market participation (Furtado & Theodoropoulos, 2010), and income (Meng & Gregory, 2005) following union formation. Considering the complex association between exogamy and cultural and structural assimilation (Rodríguez-García, 2015), we formulate a *null* hypothesis (independent of whether the woman or the man belongs to a migrant group), which forms the backdrop of our statistical analysis:

#### Assimilation hypothesis (H0)

 Fertility behaviour in exogamous unions is similar to that in endogamous native unions.

### Adaptation as a Two-Way Process

Despite this assimilation pattern, some scholars have found that fertility is higher in exogamous unions than in the respective endogamous majority group (Choi & Goldberg, 2020; Elwert, 2023; Qian & Lichter, 2021). This fertility pattern is less likely to be attributable to migrant (descendant) partners departing from cultural values (as assumed for the assimilation pattern), and is more likely be due to the preservation of different values. Research has shown that cultural assimilation may progress more slowly for fundamental life-course events, such as family formation (Carlsson, 2018). Additionally, while structural conditions can offer opportunities for inter-ethnic encounters, the migrant (descendant) partner may partially adapt to the majority group prior to or following exogamous union formation (Collet, 2015). The paths into exogamy can vary greatly, as can the potential adaptation processes between partners.

While partnering with a member of a culturally dissimilar group is associated with higher risks (e.g., loss of acceptance by the origin group), the potential partner’s traits may increase the individual gains of union formation (Alba, 2005). These traits might include similar preferences, like those for a larger family. The propensity for exogamy is relatively high among couples in which both partners are religious and have the same religious affiliation (Milewski, 2003). Religiousness is associated with a higher ideal number of children (Bein et al., 2023; Philipov & Berghammer, 2007), and may contribute to higher fertility within unions (Peri-Rotem, 2016). Moreover, religious norms might promote traditional family roles and conservative family formation patterns (McQuillan, 2004; Pearce & Thornton, 2007). Native men with migrant female partners tend to favour traditional rather than egalitarian gender roles (Glowsky, 2011), and these preferences may be associated with a stronger desire for high fertility than that of people with more egalitarian views (Kato, 2018). Furthermore, natives in exogamous unions often have more pronounced family-oriented attitudes than natives in endogamous unions (Braack & Milewski, 2019). These findings suggest that not only migrants and migrant descendants, but also natives in exogamous unions may be selected groups. While partners’ homogamy in socio-cultural traits (such as shared preferences for higher fertility, or, indirectly, sharing a religious affiliation and preferences for traditional gender roles and family solidarity) may facilitate exogamous union formation, it might also lead to these couples having higher fertility than endogamous native unions.

Qian and Lichter (2021), who found that exogamous unions’ fertility may lie between that of the respective endogamous minority and majority groups, interpreted this observation as resulting from symmetrical fertility decision-making. This again suggests the relative, or gradual, persistence of migrant (descendant)s’ fertility preferences, even in exogamous unions. Given that openness to and interest in the partners’ cultural differences may drive exogamous union formation (Singla & Holm, 2012), fertility might be higher in such unions because the partners negotiate their cultural differences through mutual adaptation (Collet, 2015). Mutual adaptation is common particularly in relation to conflicting fertility and child-timing preferences within partnerships (Thomson & Hoem, 1998). Partners negotiate their fertility preferences, which leads to a synthesis of their positions (Jansen & Liefbroer, 2006). Consequently, natives’ fertility preferences may be adapted to those of the migrant (descendant) partner, while symmetrical adjustments of exogamous unions on the micro level may result in an in-between fertility pattern on the macro level.

The abovementioned arguments challenge the traditional view that assimilation is solely a one-way process driven by migrants and their descendants. Instead, they indicate the existence of a two-way dynamic, either by underscoring the diversity among natives and questioning their role as the standard for assessing migrant assimilation, or by suggesting that assimilation at the couple level entails mutual adaptation (Alba & Nee, 2003; Klarenbeek, 2019). These insights form the basis for the first working hypothesis guiding our empirical investigation. It expands the previously observed intermarriage premium, like in the migrant’s economic assimilation (e.g., Meng & Gregory, 2005) or well-being (Milewski & Gawron, 2019; Potarca & Bernardi, 2021) on the couple level to an “intermarriage premium in family size”:

#### Intermarriage premium (H1a)

 Exogamous unions have higher fertility than endogamous native unions.

### Disruption

The previous fertility patterns indicate that exogamous unions have fertility that is similar to or higher than that of endogamous native unions. However, previous research also suggests that exogamous couples could have lower fertility than endogamous native unions (Fu, 2008; Glowsky, 2015; Qian & Lichter, 2021). From the life-course perspective, such a *disruption* may be the consequence of exogamous partnering. Exogamous unions are more likely to be higher order marriages or cohabitations (Elwert, 2020; Hohmann-Marriott & Amato, 2008). Particularly for women, pre-union fertility may have a negative impact on childbearing intentions and fertility (Stewart, 2002). Moreover, a leading factor for (higher order union) fertility is age (Beaujouan, 2020). Exogamous unions tend to occur later in life than endogamous unions (Elwert, 2020; González-Ferrer et al., 2018; Milewski, 2003; Soehl & Yahirun, 2011). Hence, the fertile phases of exogamous couples may be shorter (Schmidt et al., 2012).

Additionally, four mechanisms related to exogamous couples’ circumstances after union formation could lead to disruption. First, as they crossed ethnic boundaries in their partner choice, exogamous couples may experience discrimination by their families and a lack of familial support (Rodríguez-García et al., 2016). As family support is an important resource in childrearing, exogamous couples may need to compensate for this lack of support by devoting more time and energy to parenting (Qian & Lichter, 2021), and by spending more on non-family childcare. Thus, the increased costs of childrearing may lead these couples to have fewer children (Fu, 2008; Qian & Lichter, 2021).

Second, internal conflicts due to the partners’ cultural differences may result in fertility disruption. Divergent values and norms heighten the risk of conflicts in exogamous unions (Hohmann-Marriott & Amato, 2008; Rodríguez-García, 2006). However, differences in partners’ fertility preferences may generally contribute to the postponement of fertility decision-making (Jansen & Liefbroer, 2006). Thus, differences in fertility preferences in exogamous unions may lead partners to postpone fertility or even give up their fertility desires, thereby reducing their family size.

Third, fertility disruption may follow after the first birth. Raising children may be particularly demanding in exogamous unions because of the partners’ cultural differences (Gawron & Carol, 2022; Rodríguez-García, 2006). Partners’ experiences with their first child may affect their subsequent fertility decisions (Iacovou & Tavares, 2011). Exogamous couples may not only postpone subsequent births, but adjust (i.e., decrease) their fertility intentions more than other couples typically do, given the difficulties they face in raising children in a “mixed” family.

Fourth, the chances of exogamous couples realising their (even conflicting) fertility preferences may be lower when they experience union instability. Exogamous couples’ greater vulnerability to conflict increases their risk of union dissolution (e.g., Dribe & Lundh, 2012; Milewski & Kulu, 2014; Saarela & Finnäs, 2018). Especially unions consisting of a migrant man and a native woman tend to be less stable (Milewski & Kulu, 2014). However, initiating union dissolution may be more difficult for migrant (descendant) women than for native women (Dribe & Lundh, 2012). Moreover, exogamous unions’ potential fragility may also create poor conditions for childbearing, as childrearing requires a long-term commitment (Choi & Goldberg, 2018). Therefore, we formulate an alternative working hypothesis, in contrast to that of the intermarriage premium pattern, which focuses on the potentially disruptive effects of exogamy:

#### Disruption hypothesis (H1b)

Exogamous unions have lower fertility than endogamous native unions.

### Selection Effects

The previous considerations partly imply that the disruption, assimilation, and intermarriage premium patterns may arise from selection into exogamy (i.e., confounders). The tendency for exogamous migrant (descendant)s to have higher education levels compared to their endogamous counterparts may contribute to similar fertility rates between exogamous unions and endogamous native unions (see *assimilation pattern*). The higher fertility in exogamous unions relative to that in endogamous native unions may be attributed to exogamous partners sharing a preference for more children, sharing a religious affiliation, holding traditional views on gender roles, or valuing family solidarity, even prior to union formation (see *intermarriage premium pattern*). By contrast, risk factors related to exogamous partnering across the life course may depress fertility in exogamous unions (see *disruption pattern*).

**Fig. 2:**
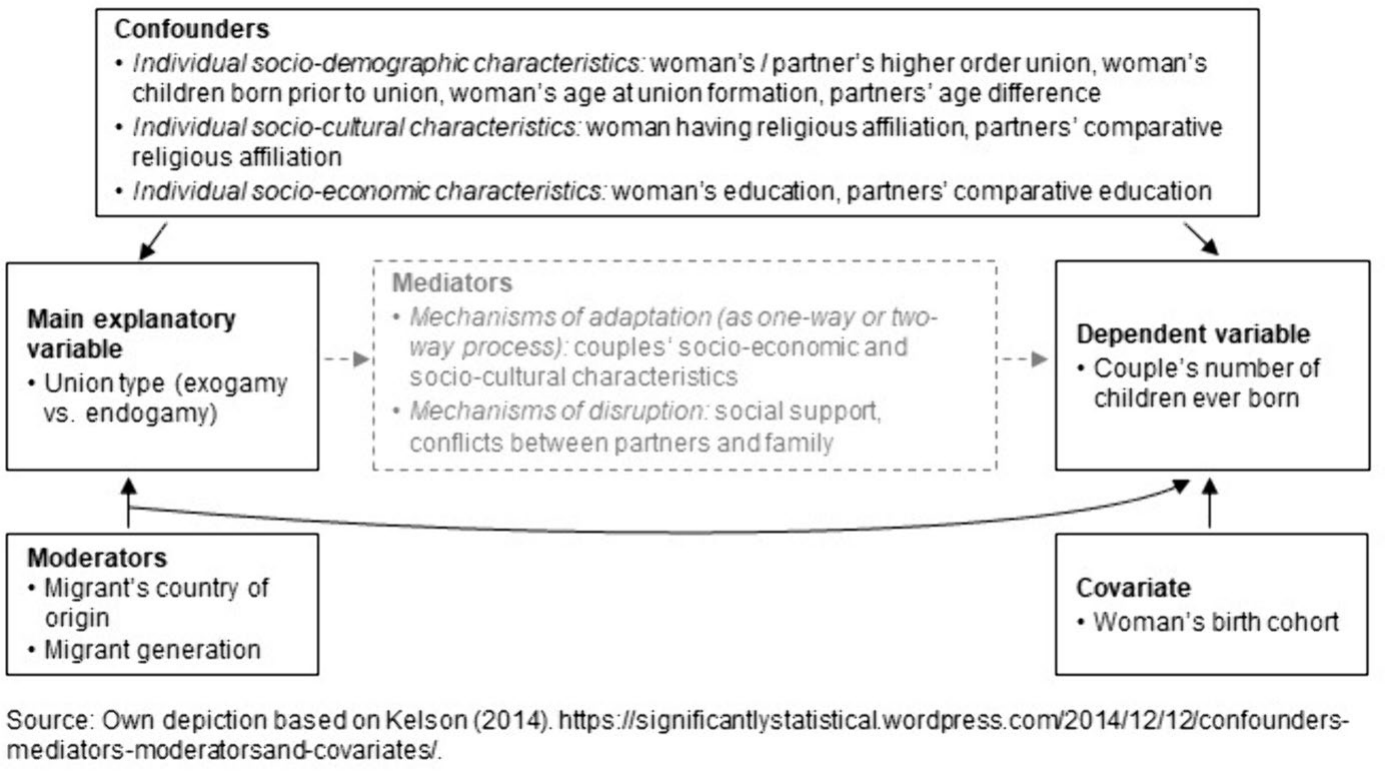
Theoretical framework for the association between exogamy and couples’ number of children ever born

When comparing fertility in exogamous unions and endogamous native unions, accounting for confounders is crucial. Beyond confounders, the link between exogamy and fertility may be influenced by a range of factors that emerge after union formation (i.e., mediators), such as social support levels, conflicts between partners and family, and socio-economic and socio-cultural adaptation processes. Similar to confounders, these mediators can have different effects on the fertility of exogamous couples, with some resulting in similar fertility and others leading to higher or lower fertility compared to that of endogamous native unions. As the link between exogamy and fertility may be mediated by these various factors, adjusting for confounders could theoretically reveal the primary influence of exogamy on fertility (Cinelli et al., 2022). However, Fig. 2 shows the confounders we can analyse with the available data. We acknowledge that we will consider only observed confounders in our investigation. Thus, we may only identify trends indicating whether exogamy could contribute to patterns of assimilation, disruption, or a premium effect concerning family size. By not including unobserved confounding variables (e.g., fertility preferences, attitudes towards gender roles, and family norms), we cannot substantiate causal claims about the observed association between exogamy and couples’ number of children ever born. To guide our analyses, we assume that the observed confounders contribute to fertility differentials between exogamous and endogamous native unions (*selection hypothesis*, H2).

### Differences by the Migrant Partner’s Country of Origin and Generation

This section considers the *moderating* role of the migrant (descendant)’s country of origin and generation. Considering the role of socialisation, exogamous unions with migrant (descendant) partners from culturally less similar countries with higher fertility than Germany (e.g., countries in Africa or the Middle East, Adserà & Ferrer, 2016) are especially likely to have larger families than the endogamous majority group. Moreover, migrant descendants may have higher fertility than natives as “changes in fertility preferences might take more than one generation” (Adserà & Ferrer, 2015, p. 362). However, the fertility differences between exogamous unions and endogamous native unions may be smaller if the exogamous unions involve migrant descendants rather than first-generation migrants, as adjustments to the majority group’s fertility values, norms, and preferences occur across generations (Kulu et al., 2019).

US findings underscore the importance of considering ethnic and cultural differences within exogamous couples. Because the US has a longer history of ethnic diversity than Western Europe, where the formation of new ethnic minorities did not begin decisively until after World War II, US studies focus less on the differentiation between natives, migrants, and migrant descendants (which is common in European investigations). US research refers instead to ethnic or racial categories. Based on these categories, scholars observed for the US context that especially Black–White pairings tend to have higher fertility than endogamous unions of the majority group (Choi & Goldberg, 2020; Qian & Lichter, 2021). In the US, boundaries between Blacks and Whites are perceived as being strong (Alba, 2005). Strong boundaries (i.e., larger ethnic differences) are harder to cross than smaller ethnic differences, even in the context of exogamy (Qian & Lichter, 2007). In Germany, where religion serves as a boundary marker (Alba, 2005), unions between natives and, for instance, migrant descendants from Catholic countries like Spain and Italy are far more common than unions between natives and individuals of Turkish origin (Schroedter & Kalter, 2008).

We assume that in Europe, similar to in the US, the fertility rates of unions in which the social and cultural differences between the partners are larger may differ from those of more typical exogamous unions (like unions between descendants of European migrants and natives). This variation can be attributed not only to the migrant (descendant)’s socialisation and generational status, but also to the incentives linked to the formation of such unions. Given the role of homogamy in union formation (Kalmijn, 1998), similarity in traits other than ethnicity becomes particularly relevant for establishing *less likely* exogamous unions. We previously highlighted the role of socio-cultural confounders, like the partners’ shared fertility preferences, religious beliefs, and attitudes towards family and gender roles. These traits may foster exogamous union formation, especially among partners with greater cultural and social diversity, potentially boosting fertility in these unions. Regarding the two moderators of country of origin and migrant generation, we assume that exogamous unions of partners who are less socially and culturally similar (in terms of country of origin and migrant generation) have higher fertility than exogamous unions of more similar partners (*subgroup hypothesis*, H3).

## Methodology

### The Data

This study uses data from the GSOEP (Wagner et al., 2007; DOI info: 10.5684/soep.core.v37eu), a yearly panel study that started in 1984 as a random representative sample of private households in West Germany and was extended in 1990 to cover East Germany. The GSOEP oversamples migrants; initially from the five quantitatively most important countries of origin from which so-called “guest workers” were recruited (Turkey, Greece, Then-Yugoslavia, Italy, and Spain). Additional samples were selected to augment the migrant population, especially during periods of heightened immigration (Goebel et al., 2019). We use data from 37 waves up to 2020 in the SOEP long format in order to consider a broad sample of natives and (first-, 1.5-, and second-generation) migrants. This study defines a *native* as an individual who was born in Germany and whose parents were born in Germany. A *migrant (descendant)* is defined as an individual who was either born outside of Germany or was born in Germany to two parents who were born abroad. To determine the country of origin for migrant descendants born in Germany, we utilize their mothers’ country of origin. Note that migrant descendants born in Germany to one native and one migrant parent are excluded from this investigation.

To analyse differences by union type in couples’ number of children ever born, we decompose women’s completed fertility by their union history. Corresponding to the cohort fertility rate, we identify women’s completed fertility at age 40 (i.e., we do not consider births after age 40). This threshold is suitable for empirical analyses, since only 1.5% of the women in our sample reported giving birth after age 40. Moreover, we focus on women’s first and (when applicable) second non-marital or marital cohabitation during their reproductive years (as in our sample, fertility mainly occurred in these two union types). We only analyse opposite-sex unions formed by women up to age 39. Right-censored cases in which we lack information about the woman’s completed fertility at age 40, or in which the woman did not mention partnerships up to age 39, are excluded from the sample.

To ensure that all unions in our sample face similar societal conditions, such as similar relative opportunity costs of childbirth, we delete (exogamous and endogamous) couples involving migrants when the unions were formed more than one year before the migrant’s move to Germany. Moreover, we exclude unions with migrants who immigrated after age 30 to avoid underestimating fertility. To further decrease the heterogeneity of the sample, we only select unions in which the women were born between 1940 and 1980. All in all, our analytical sample consists of 7218 couples. The majority are married with an average union duration of 15.2 years (see Supplementary Table S-1.1). Supplementary Table S-1.2 illustrates the sample structure by the order of the woman’s union. Overall, in only 18 out of 165 cases of second unions, we notice a different union type than that of the preceding first union (i.e., the second union is exogamous while the first union is endogamous, or vice versa).

### Operationalisation

We obtain the *couples’ number of children ever born* by merging all available childbirth-related data of women up to age 40. Based on each woman’s first two unions, we count her number of births from the beginning to the end of each union’s observation time. The union’s observation time starts with the year of the couple’s joint household formation or with the year of marriage (when information about previous premarital cohabitation within unions that transitioned into marriages is unavailable). The union’s observation time ends when the woman reached age 40. If separation, divorce, or widowhood occurred before the woman turned 40, the observation is restricted by the year of union dissolution. To reduce the impact of extreme values that skew the estimates, we winsorize couples’ number of children ever born at the 99.9th percentile (six cases in total with seventh, eighth, and 10th births), which leads to a distribution from zero to six births.

Our main explanatory variable is union type, which distinguishes between endogamous and exogamous partnerships based on the woman’s and her partner’s migrant status and country of origin. In exogamous unions, one partner is a native and the other partner is a migrant (descendant). If the partners reported having the same country of origin, their union type is labelled as endogamous (unions in which both partners are migrants or migrant descendants but from different origins, representing 100 cases in total, are omitted from the sample). This results in four *union type groups*: endogamous native (87.8%) and endogamous migrant (descendant) unions (6.9%), and exogamous unions with native women (2.5%) and migrant (descendant) women (2.8%). For our sample, Table 1 shows the five most frequent countries of origin by union type among the migrant (descendant) partners in this study. The outlined shares mirror the varying frequencies of different compositions of ethnically mixed unions in Germany (Schroedter & Kalter, 2008).

**Table 1:** Migrant (descendant)s’ most frequent countries of origin in the analysis sample, by union type. *Source:* Calculations based on GSOEP 1984–2020, N = 882

Five most frequent countries of origin	Exogamous unions with native women			Exogamous unions with migrant (descendant) women			Endogamous unions of migrant (descendant)s		
	Men’s countries of origin	%	n	Women’s countries of origin	%	n	Women’s countries of origin	%	n
1	South Europe	34.3	62	North/West Europe + English speaking countries	33.5	67	MENA	33.9	170
2	North/West Europe + English speaking countries	22.1	40	East Europe	19.0	38	South Europe	32.7	164
3	East Europe	12.7	23	Asia	16.5	33	South-East Europe	14.6	73
4	MENA	11.6	21	South-East Europe	9.0	18	East Europe	9.4	47
5	South-East Europe	9.4	17	South Europe	8.5	17	Asia	7.5	37
Other countries	–	9.9	18	–	13.5	27	–	2.0	10
*Total*		*20.5*	*181*		*22.7*	*200*		*56.8*	*501*

*Asia*: Armenia, Belarus, China, Georgia, India, Japan, Kazakhstan, Laos, Philippines, Russia, Sri Lanka, Thailand, Ukraine, Uzbekistan, Vietnam; *East Europe*: Czech Republic, Hungary, Lithuania, Poland, Slovakia, Slovenia; *North/West Europe* + *English speaking countries*: Austria, Belgium, Canada, Denmark, Finland, France, Great Britain, Ireland, Sweden, Switzerland, The Netherlands, United States; *Middle East and North Africa (MENA)*: Afghanistan, Egypt, Iran, Iraq, Lebanon, Morocco, Palestine, Syria, Tunisia, Turkey; *South Europe*: Greece, Italy, Portugal, Spain; *South-East Europe*: Bosnia-Herzegovina, Bulgaria, Croatia, Kosovo, Macedonia, Romania, Serbia, Then-Yugoslavia

Except for our covariate of the *woman’s year of birth,* the following characteristics are separately built for each analysed union when more than one union is reported for a person. *Migrant generation* distinguishes between first-generation migrants (immigrated at age 16 or older) and migrant descendants (immigrated at age 15 or younger, or born in Germany). Additionally, we identify each *country of origin* of migrants and their descendants as a high- or low-fertility region. The variable is based on the total fertility rate (TFR) of the country of origin. To account for the broad range of women’s birth years in our sample (1940–1980), we calculate for each country of origin a mean TFR from 1960 to 2020 based on World Bank Data (2023). We define regions with mean TFRs above 2.1 as high-fertility, and regions with mean TFRs of 2.1 and below as low-fertility (see Supplementary Table S-1.3, which shows the countries of the two groups in detail).

Socio-demographic confounders: Two dummies capture separately for women and their partners whether the analysed union is their first union or a *higher order union*. Moreover, we include a dummy measuring whether the woman reported no births or at least one *birth prior to union formation*. Note that we would have ideally accounted for the male partner’s previous children, too. Unfortunately, the GSOEP did not start collecting male respondents’ fertility histories until 2001, which would provide information for only 54% of our sample. Other variables cover the *woman’s age at union formation* and *the relative age difference between the partners*. The latter is categorised into three groups: similar age (the woman is no more than one year older or up to five years younger than the man), the woman is significantly younger (the woman is six or more years younger than the man), or the woman is older (the woman is at least two years older than the man).

Socio-cultural and socio-economic confounders: A dummy captures whether the woman does or does not *have a religious affiliation*. The variable *partners’ comparative religious affiliation* indicates whether the partner has the same religious affiliation as the woman, measured by distinguishing between different denominations (e.g., Catholicism, Protestantism, Islam). Moreover, the *woman’s education* is measured by ISCED (1997) levels grouped into the following categories: low (levels 0–2), upper secondary (levels 3–4), and tertiary education (levels 5–6). The *partners’ comparative education* is also measured using ISCED (1997) codes. The variable shows that both partners have the same education (homogamy), or that the woman has lower (hypergamy) or higher education than her partner (hypogamy). Note that for the socio-cultural and socio-economic confounders, we use the woman’s and her partner’s values for each characteristic at the time of union formation (i.e., in the year of union formation or earlier). Missing values for a characteristic occur when the woman and her partner were only interviewed during or after the union’s observation time. To diminish the number of missing values, we add the earliest values after union formation. As this information is not always available, not all missing values can be replaced.

### Methods

We apply generalized Poisson regression models (Harris et al., 2012; Wang & Famoye, 1997). This approach considers the couples’ number of children ever born as a count variable, while the given data are under-dispersed. To consider repeated measures on individuals, we use clustered standard errors. Moreover, we use multiple imputation with chained equations (m = 20) to replace missing values in four variables (see Table 2).

**Table 2:** Descriptive statistics, by union type and women’s migrant status. *Source:* Calculations based on GSOEP 1984−2020, N = 7218

	Min	Max	Exogamous unions with …				Endogamous unions with …			
			native women		migrant (descendant) women		native women		migrant (descendant) women	
			Mean/ %	SD/ n	Mean/ %	SD/ n	Mean/ %	SD/ n	Mean/ %	SD/ n
*Dependent variable*										
Couple’s number of children ever born in union (metric^**†**^)	0	6	1.6	1.1	1.8	1.1	1.7	1.0	2.2	1.1
0	–	–	18.2	33	13.5	27	14.6	926	7.2	36
1	–	–	24.3	44	22.5	45	23.8	1505	13.4	67
2	–	–	38.1	69	42.5	85	43.3	2743	45.1	226
3 +	–	–	19.3	35	21.5	43	18.3	1162	34.3	172
*Confounders*										
Woman’s year of birth (metric)	1940	1980	1962	9.5	1962	11.1	1957	10.2	1960	11.5
Woman’s age at union formation (metric)	16	39	25.8	4.8	26.5	4.5	24.4	4.9	22.8	4.5
Partner’s age difference										
Woman is ≤ 1 year older / ≤ 5 years younger than man (woman’s = man’s)	–	–	64.1	116	58.5	117	73.6	4665	58.7	294
Woman ≥ 6 years younger than man (woman’s < man’s)	–	–	16.6	30	34.5	69	19.1	1212	33.1	166
Woman ≥ 2 years older than man (woman’s > man’s)	–	–	19.3	35	7.0	14	7.2	459	8.2	41
Woman’s children born prior to union										
No	–	–	88.4	160	87.0	174	88.2	5587	93.2	467
Yes	–	–	11.6	21	13.0	26	11.8	749	6.8	34
Woman’s order of union										
First	–	–	95.6	173	98.0	196	97.6	6181	99.8	500
Higher	–	–	4.4	8	2.0	4	2.4	155	0.2	1
Partner’s order of union										
First	–	–	93.9	170	88.5	177	93.2	5906	96.0	481
Higher	–	–	6.1	11	11.5	23	6.8	430	4.0	20
Woman’s education										
None, primary, lower secondary	–	–	13.8	25	16.5	33	10.1	640	51.3	257
Upper secondary	–	–	56.4	102	45.0	90	62.6	3967	40.5	203
Tertiary	–	–	29.3	53	38.0	76	26.9	1704	7.6	38
Mv	–	–	0.6	1	0.5	1	0.4	25	0.6	3
Partner’s comparative education										
Homogamy (woman’s = man’s)	–	–	43.6	79	44.0	88	53.7	3405	41.9	210
Hypergamy (woman’s < man’s)	–	–	29.3	53	33.5	67	30.3	1918	33.7	169
Hypogamy (woman’s > man’s)	–	–	26.5	48	21.0	42	14.6	923	23.4	117
Mv	–	–	0.6	1	1.5	3	1.4	90	1.0	5
Woman having religious affiliation										
No	–	–	15.5	28	20.0	40	25.4	1611	7.0	35
Yes	–	–	75.1	136	73.5	147	63.2	4002	82.4	413
Mv	–	–	9.4	17	6.5	13	11.4	723	10.6	53
Partner’s comparative religious affiliation										
Homogamy (woman’s = man’s)	–	–	42.0	76	49.0	98	61.8	3913	77.0	386
Heterogamy (woman’s ≠ man’s)	–	–	44.8	81	42.0	84	24.0	1518	11.0	55
Mv	–	–	13.3	24	9.0	18	14.3	905	12.0	60
*Moderators*										
Migrants’ region of origin										
Low-fertility countries	–	–	75.1	136	74.5	149	n.a	–	56.3	282
High(er)-fertility countries	–	–	24.9	45	25.5	51	n.a	–	43.7	219
Migrant generation										
First-generation migrants (G1)	–	–	49.2	89	67.5	135	n.a	–	75.6	379
Migrant descendants (G1.5 + G2)	–	–	50.8	92	32.5	65	n.a	–	24.4	122
*Total*	–	–	*2.5*	*181*	*2.8*	*200*	*87.8*	*6336*	*6.9*	*501*

*mv* = missing values, *n.a.* = not applicable

^†^mean is based on the birth distribution from 0 to 6 children

The multivariable analysis is carried out in a stepwise fashion. Model 1 examines couples’ fertility by union type and the woman’s birth year. Model 2 introduces socio-demographic confounders (order of the analysed union, the woman’s pre-union fertility, the woman’s age at union formation, and the partners’ age differences). Model 3 considers socio-cultural and socio-economic characteristics, including the woman’s religious affiliation, the partners’ religious heterogamy, the woman’s education, and the partners’ educational differences. In further analyses, we repeat the examination by distinguishing between unions with migrants from low- and high-fertility regions, and unions with migrants and migrant descendants.

## Results

### Exogamy and Couples’ Number of Children Ever Born

Descriptively, we observe differences in couples’ number of children ever born by union type (see Table 2). The mean number of children is 2.2 for endogamous migrant (descendant) unions and is 1.6 for exogamous unions with native women. Average fertility in exogamous unions with native women is slightly lower than that in exogamous unions with migrant (descendant) women, at around 1.8 children, and in endogamous native unions, at around 1.7 children. Including the woman’s birth year in *Model 1/*Table 3 results in no fertility differences between exogamous and endogamous unions with native women. However, exogamous unions with migrant (descendant) women have higher fertility than endogamous native unions, with statistical significance at a 10% level. Thus, our findings suggest an assimilation pattern among exogamous unions with native women, and an intermarriage premium in family size among exogamous unions with migrant (descendant) women. Next, we control for whether the fertility patterns hold after accounting for confounders.

**Table 3:** Determinants of couples’ number of children ever born. *Source:* Calculations based on GSOEP 1984–2020

	M1		M2		M3	
	Coef	(S.E.)	Coef	(S.E.)	Coef	(S.E.)
Union type						
Endogamous: native women	Ref		Ref		Ref	
Exogamous: native women	− 0.00	(0.05)	0.00	(0.04)	0.01	(0.05)
Exogamous: migrant (descendant) women	0.09^+^	(0.05)	0.14***	(0.04)	0.12**	(0.04)
Endogamous: migrant (descendant) women	0.25***	(0.02)	0.14***	(0.02)	0.10***	(0.03)
Woman’s year of birth (mean centered)	− 0.00***	(0.00)	0.00***	(0.00)	0.00***	(0.00)
Woman’s age at union formation (mean centered)			− 0.04***	(0.00)	− 0.04***	(0.00)
Partner’s age difference						
Woman is ≤ 1 year older / ≤ 5 years younger than man (woman’s = man’s)			Ref		Ref	
Woman ≥ 6 years younger than man (woman’s < man’s)			0.00	(0.02)	− 0.00	(0.02)
Woman ≥ 2 years older than man (woman’s > man’s)			0.11***	(0.03)	0.10**	(0.03)
Woman’s order of union						
First			Ref		Ref	
Higher			− 0.37***	(0.06)	− 0.37***	(0.06)
Partner’s order of union						
First			Ref		Ref	
Higher			− 0.21***	(0.04)	− 0.16***	(0.04)
Woman’s children born prior to union						
No			Ref		Ref	
Yes			− 0.33***	(0.03)	− 0.26***	(0.03)
Woman’s education						
None, primary, lower secondary					Ref	
Upper secondary					− 0.06**	(0.02)
Tertiary					0.08**	(0.03)
Partner’s comparative education						
Homogamy (woman’s = man’s)					Ref	
Hypergamy (woman’s < man’s)					0.03	(0.02)
Hypogamy (woman’s > man’s)					− 0.10***	(0.02)
Woman having religious affiliation						
No					Ref	
Yes					0.21***	(0.02)
Partner’s comparative religious affiliation						
Homogamy (woman’s = man’s)					Ref	
Heterogamy (woman’s ≠ man’s)					− 0.06***	(0.02)
*Constant*	0.53***	*(0.01)*	0.55***	*(0.01)*	0.43***	*(0.03)*
*N*	*7218*		*7218*		*7218*	

Standard errors in parentheses, ^+^p < 0.10, *p < 0.05, **p < 0.01, ***p < 0.001

Accounting for socio-demographic confounders in *Model 2/*Table 3, the fertility of exogamous unions with native women remains comparable to that of endogamous native unions, although the coefficient shifts from negative to positive. Furthermore, in Model 2, exogamous unions involving migrant (descendant) women have fertility rates similar to those of endogamous migrant (descendant) unions, exceeding the fertility rates of endogamous native unions with a significance level of 0.1%. The changes in the coefficients for both groups of exogamous unions can particularly be attributed to the inclusion of the woman’s age at union formation. Exogamous migrant (descendant) women tend to have the highest mean age at union formation, followed by exogamous native women (see Table 2), which correlates with fewer childbirths (see Table 3). However, considering socio-demographic confounders only results in a more pronounced intermarriage premium pattern among exogamous unions with migrant (descendant) women, whereas the fertility pattern of exogamous unions with native women continues to suggest assimilation.

In Model 3 (Table 3), which introduces socio-economic and socio-cultural variables, the previously observed fertility patterns are maintained, with slight coefficient changes in exogamous unions, mainly due to the inclusion of the woman’s religious affiliation. Both native and migrant (descendant) women in exogamous unions are more likely than those in endogamous native unions to have a religious affiliation (see Table 2), which is linked to higher fertility (see Table 3). Adding the religion variable diminishes the coefficient for exogamous unions with migrant (descendant) women, but enhances it for those with native women. Despite the adjustments in Model 3 (Table 3), the fertility pattern of exogamous unions with native women still indicates assimilation, and that of migrant (descendant) women still points to the intermarriage premium.

In summary, our analysis reveals fertility differences between endogamous native and migrant (descendant) unions, with the latter generally exhibiting higher fertility. However, our findings for exogamous unions vary. First, while no statistically significant *fertility disruptions *(H1b) are observed, a negative coefficient in the first model suggests potential disruptions, particularly in unions involving native women. Second, the results for exogamous unions involving native women consistently support the *assimilation hypothesis* (H0). Conversely, exogamous unions with migrant (descendant) women exhibit higher fertility than endogamous native unions, especially in Models 2 and 3, in line with our *intermarriage premium hypothesis* (H1a). Third, we find support for *selection* (H2), with differing effects for the two groups of exogamous unions. Especially in the case of exogamous unions with migrant (descendant) women, the intermarriage premium becomes more pronounced after considering life-course transitions, particularly women’s older ages at union formation. Additionally, the intermarriage premium of exogamous unions with migrant (descendant) women is partly explained by religious affiliation. Even though migrant (descendant) and native women in exogamous unions exhibit similar patterns of religious affiliation, fertility in exogamous unions with native women does not decrease when accounting for the woman’s religious affiliation. Thus, the association between religious norms (which often advocate for traditional family roles and conservative family formation patterns) and fertility seems less pronounced in unions of native women/migrant (descendant) men than in unions of migrant (descendant) women/native men.

### Differences by the Migrant’s Country of Origin and Generation

Next, we examine differences in fertility patterns across union type groups, considering the migrant (descendant)s’ region of origin and migrant generation. Table 4, *subgroup analysis A*, presents the coefficients of the union type groups, distinguishing by different regions of origin. We first focus on high-fertility regions. From Model 2 (Table 4) onwards, the fertility of exogamous unions involving women from high-fertility regions clearly exceeds that of endogamous native unions, resembling the fertility levels of endogamous migrant (descendant) unions. Additionally, the coefficient for unions of native women/men from high-fertility regions falls between those for endogamous native and migrant (descendant) unions from Model 2 onwards. However, due to statistical significance, we can only regard the finding on exogamous unions with women from high-fertility regions as evidence of an intermarriage premium.

**Table 4:** Determinants of couples’ number of children ever born, by migrant (descendant)s’ region of origin and migrant generation. *Source:* Calculations based on GSOEP 1984–2020

Subgroup analysis	M1		M2		M3	
	Coef	(S.E.)	Coef	(S.E.)	Coef	(S.E.)
*(A) Migrant (descendant)s’ region of origin:*						
Union type # migrant (descendant)s’ region of origin						
Endogamous: native women	Ref		Ref		Ref	
Migrants from high-fertility regions						
Exogamous: native women	0.02	(0.09)	0.09	(0.08)	0.12	(0.08)
Exogamous: migrant (descendant) women	0.10	(0.11)	0.22*	(0.10)	0.17^+^	(0.10)
Endogamous: migrant (descendant) women	0.35***	(0.04)	0.21***	(0.03)	0.18***	(0.04)
Migrants from low-fertility regions						
Exogamous: native women	− 0.01	(0.06)	− 0.03	(0.05)	− 0.02	(0.05)
Exogamous: migrant (descendant) women	0.08^+^	(0.05)	0.11*	(0.05)	0.10*	(0.05)
Endogamous: migrant (descendant) women	0.15***	(0.03)	0.07*	(0.03)	0.02	(0.03)
*Constant*	0.53***	*(0.01)*	0.55***	*(0.01)*	0.43***	*(0.03)*
*N*	*7218*		*7218*		*7218*	
*(B) Migrant generation:*						
Union type # migrant generation						
Endogamous: native women	Ref		Ref		Ref	
First-generation migrants						
Exogamous: native women	0.00	(0.07)	0.01	(0.07)	0.02	(0.07)
Exogamous: migrant (descendant) women	0.11*	(0.05)	0.16***	(0.05)	0.15**	(0.05)
Endogamous: migrant (descendant) women	0.25***	(0.03)	0.15***	(0.03)	0.12***	(0.03)
Migrant descendants						
Exogamous: native women	− 0.01	(0.06)	− 0.01	(0.06)	0.00	(0.06)
Exogamous: migrant (descendant) women	0.04	(0.09)	0.11	(0.09)	0.07	(0.08)
Endogamous: migrant (descendant) women	0.23***	(0.05)	0.09*	(0.04)	0.05	(0.05)
*Constant*	0.53***	*(0.01)*	0.55***	*(0.01)*	0.43***	*(0.03)*
*N*	*7218*		*7218*		*7218*	

Standard errors in parentheses, ^+^*p* < 0.10, *p < 0.05, **p < 0.01, ***p < 0.001

M1 = +woman’s year of birth

M2 = +woman’s and partner’s higher order union, woman’s children born prior to union, woman’s age at union formation, partner’s age difference

M3 = +woman having religious affiliation, partner’s comparative religious affiliation, woman’s education, partner’s comparative education

Focusing on low-fertility regions shows that differences between endogamous native and migrant (descendant) couples vanish by Model 3 (Table 4). Correspondingly, in Model 3, we see assimilation patterns among exogamous unions with native women and endogamous migrant (descendant) unions. However, exogamous unions with migrant (descendant) women from low-fertility regions display higher fertility than endogamous native unions. Thus, unions of migrant (descendant) women/native men have a larger family size, regardless of origin. Nevertheless, exogamous unions with female and male partners from high-fertility regions consistently have higher fertility than those with migrant (descendant) partners from low-fertility regions.

*Subgroup analysis B* (Table 4) distinguishes between unions involving first-generation migrants and unions involving migrant descendants. The fertility rates of unions with first-generation migrant women, whether endogamous or exogamous, are higher than those of endogamous native unions. While exogamous and endogamous unions with female migrant descendants also have higher fertility than endogamous native unions, the differences are not statistically significant, suggesting an assimilation pattern. Exogamous unions with native women exhibit an assimilation pattern, regardless of the partner’s generational status. However, exogamous unions involving female and male first-generation migrants generally have higher fertility than those with migrant descendants.

In summary, the fertility differences between the two groups of endogamous unions are most pronounced when comparing natives with migrant (descendant)s from high-fertility regions, and are notable, albeit less pronounced, when comparing natives with first-generation migrants. Regarding exogamous unions with native women, our findings across all subgroup analyses consistently support the *assimilation hypothesis* (H0), although some of the results for unions of native women/men from high-fertility regions suggest an intermarriage premium. Conversely, exogamous unions with migrant (descendant) women show an *intermarriage premium *(H1a) pattern across most subgroup analyses. This pattern is not statistically significant only in unions involving female migrant descendants. Despite the varied fertility patterns, Table 4 supports the *subgroup hypothesis* (H3) for all exogamous unions.

## Discussion

By examining the association between couples’ number of children ever born and exogamy, our paper analyses two measures of migrant assimilation: partner choice and fertility. The empirical analyses have produced rich material, and we conclude by highlighting the main findings. First, the number of children in exogamous unions exceeds that in endogamous native couples, as observed in exogamous unions with first-generation migrant women, and in those with women from both low- and high-fertility regions. Additionally, our results suggest that fertility is higher in exogamous unions with female migrant descendants and in exogamous unions between native women and migrant men from high-fertility countries, although these findings are not statistically significant. In particular, the finding of statistically significant higher fertility in exogamous unions than in endogamous native unions supports the hypothesis of an *intermarriage premium in family size* (H1a). This implies a two-way character of assimilation within exogamous unions (Alba & Nee, 2003; Klarenbeek, 2019). As we cannot adequately account for socio-cultural confounders, whether motivations for union formation—such as both partners having a preference for higher fertility or favouring traditional gender roles and family solidarity before union formation—contribute to the intermarriage premium in family size remains unclear. Additionally, whether alignment in the partners’ values and preferences after union formation plays a role is uncertain. To answer such questions, more research is needed on values, preferences, and attitudes regarding fertility and family among exogamous natives, migrants, and migrant descendants (see also Braack & Milewski, 2019). Specifically, addressing the impact of exogamy on changes in socio-cultural factors, as was previously done for exogamy and well-being (Potarca & Bernardi, 2021) or migrants’ structural assimilation (Dribe & Nystedt, 2015), will provide further insights into assimilation processes at the micro societal level and the link between the two analysed indicators of migrant assimilation.

Second, our results point to gendered patterns of assimilation. Although we do not distinguish between exogamous unions with native or with migrant (descendant) women in our hypotheses, our findings reveal that exogamy has different implications depending on whether the female or the male partner is a migrant (descendant). We find that exogamous unions with native women have lower fertility than exogamous unions with migrant (descendant) women. More precisely, after accounting for *selection into exogamy* (H2), we observe an *intermarriage premium in family size* (H1a) among exogamous unions with migrant (descendant) women. In contrast, exogamous unions with native women show an *assimilation fertility pattern* (H0), both before and after accounting for selection into exogamy. The observed differences in fertility between exogamous unions with native women and those with migrant (descendant) women are in line with previous research that found differences depending on exogamous couples’ gender composition. Overall, exogamous couples with native women seem to be more disadvantaged than exogamous couples with native men in terms of their risk of poor mental health (Eibich & Liu, 2021; Milewski & Gawron, 2019) or of divorce (Milewski & Kulu, 2014). The latter finding aligns with our observation that the shortest union durations are in exogamous unions with native woman (see Supplementary Table S-1.1). Moreover, Braack et al. (2021) demonstrated that natives in exogamous unions are more likely than their endogamous counterparts to be the main earner. It seems that exogamous unions involving native women are more likely to have precarious household conditions, and therefore tend to face higher opportunity costs for childbearing, than exogamous unions involving migrant (descendant) women. These different opportunity costs may contribute to the observed differences between exogamous unions with native women and those with migrant (descendant) women. Despite the different fertility conditions in exogamous unions with native women, we do not find a *disruption* (H1b) in their number of children. However, as our analyses are conducted in the low-fertility context of Germany, the question of whether a disruption in family size would be observed among exogamous unions with native women in destination countries with higher fertility remains open.

While relatively minor differences by union type are found after considering confounders, and although confounding effects cannot be entirely eliminated, we conclude that exogamy seems to influence fertility. However, the implications of the union type particularly depend on the gender of the migrant (descendant) partner. The obtained findings highlight the relevance of including information on both partners’ migrant status in analyses on fertility, in accordance with previous research on fertility decision-making that emphasised the roles of both partners (Bauer & Kneip, 2013, 2014) and of migrant (descendant) men (Milewski & Baykara-Krumme, 2023). Moreover, the results suggest a two-way character of assimilation within exogamous unions, raise questions regarding the causal link between exogamy and socio-cultural factors, and indicate that the couple’s employment patterns in particular may mediate the association between exogamy and family size.

Lastly, the findings indicate how exogamy may drive social change within the mainstream society (Alba, 2022). Exogamous unions do not have fewer children than endogamous native unions; rather, bigger family sizes in exogamous unions with migrant (descendant) women may contribute to the growth of the multi-ethnic population. While the proportion of exogamous unions is small, the number increases across generations (see Table 2). Previous research suggests that migrant descendants, particularly from certain non-Western groups, have higher fertility than the native population (Kulu & González-Ferrer, 2014). Furthermore, we note that exogamous unions involving female migrant descendants tend to have higher fertility than endogamous native unions. Therefore, a detailed analysis of fertility patterns in exogamous unions with migrant descendants by origin could provide further insights into how exogamy influences social change within mainstream society.

Like others, this study is not without limitations. Our study has a descriptive character. The smaller case numbers for exogamous unions allowed for only rough categorisations of the migrant (descendant)’s country of origin and migrant generation. As the share of minorities is growing in the population, future research will benefit from the ability to distinguish more precisely between origin groups. Additionally, due to the limited information on men’s number of children before union formation, future research should explore how this confounder affects exogamous couples’ fertility. This study also raises questions about how individuals in exogamous unions differ from those in endogamous unions in terms of socio-cultural traits like fertility preferences and family and gender role attitudes prior to and after union formation. To gain a deeper understanding of how exogamy affects fertility, future research should examine how couples’ employment patterns influence the relationship between exogamy and fertility. Due to insufficient data, this study could not explore mediating factors (like couples’ employment patterns, social support, partner and family conflicts). However, the relationship between these mediating variables and couples’ fertility is particularly complex when focusing on couples’ number of children ever born. For example, while the birth of a couple’s first child can change their employment patterns, the first birth may be influenced by exogamy, and can, in turn, affect the likelihood of subsequent births (“mediator-outcome confounding influenced by the exposure”, Richiardi et al., 2013, pp. 1516). Thus, an initial step may be to consider these variables in studies focusing on first-birth transitions.

Despite some limitations, our results are partly in line with previous research on exogamy and fertility. Research on fertility intentions also highlighted the role of selection into exogamy (Pereiro et al., 2023). However, we observe some differences as well. Contrary to our findings, an investigation on fertility intentions (Pereiro et al., 2023) supported the assimilation hypothesis for exogamous migrant women after accounting for exogamous couples’ selectivity. Moreover, whereas studies on other contexts, like the US (Choi & Goldberg, 2018; Qian & Lichter, 2021) and the Swedish context (Elwert, 2023), reported higher fertility in exogamous unions with minority group men, our findings show larger family sizes in exogamous unions with migrant women. It is possible that deviations from Elwert’s results (2023) are attributable to sample selectivity. Elwert (2023) analysed third-birth risks, which may heighten sample selectivity. This is primarily because the investigated women already have two children, and it is especially relevant among exogamous couples with native women due to the shorter duration of these relationships (see Supplementary Table S-1.1). Overall, future investigations of couples’ number of children ever born could clarify whether differences in fertility measures, samples, or minority population compositions contribute to these varied findings.

We are identifying a further avenue for research. The observed fertility differences between exogamous unions involving native and migrant (descendant) women raise questions regarding offspring in mixed families. As the experiences of growing up in these unions may differ too, future research on mixed children’s life chances should consider the different constellations of exogamous parents in relation not only to origin (e.g., Irastorza & Elwert, 2021), but also to gender.

By combining the two research streams of partner choice and fertility, our study has provided new insights into ethnically diverse societies. As this is the first investigation that has focused on couples’ number of children ever born by considering the different union types of and between migrant (descendant)s, and natives, our results have relevance for studies in migration research and family sociology. The findings challenge existing assimilation theories, which are often gender-blind, omit natives, and expect migrants and migrant descendants in exogamous unions to be adapting to the majority society in their country of destination per se. Meanwhile, the lived reality is more complex (Baykara-Krumme, 2023).

## Supplementary Information

Below is the link to the electronic supplementary material.

Supplementary file1 (DOCX 59 KB)
