# Supplementary material for: Migration, Partner Selection, and Fertility in Germany: How Many Children are Born in Mixed Unions?
Source: Eur J Popul. 2024 Jun 28;40(1):24. doi: 10.1007/s10680-024-09710-w (PMC11213842; doi:10.1007/s10680-024-09710-w)
Supplement: Supplementary file 1 — Supplementary file1 (DOCX 59 KB) [file 10680_2024_9710_MOESM1_ESM.docx]

# **Supplementary for the article**

*“Migration, partner selection, and fertility in Germany:*

*How many children are born in mixed unions?”*

# **1 Additional Descriptive Statistics**

**Table S-1.1: Characteristics of the unions**

|  | | | ***Exogamous unions with …*** | | | |  | ***Endogamous unions with …*** | | | |  | ***Total*** | |
| --- | --- | --- | --- | --- | --- | --- | --- | --- | --- | --- | --- | --- | --- | --- |
|  | | | ***native***  ***women*** | | ***migrant (descendant) women*** | |  | ***native***  ***women*** | | ***migrant (descendant) women*** | |  |  |  |
|  | Min | Max | Mean/  % | SD/  n | Mean/  % | SD/  n |  | Mean/  % | SD/  n | Mean/  % | SD/  n |  | Mean/  % | SD/  n |
|  |  |  |  |  |  |  |  |  |  |  |  |  |  |  |
| *Union’s institutional degree* |  |  |  |  |  |  |  |  |  |  |  |  |  |  |
| Marriage | - | - | 91.2 | 165 | 98.5 | 197 |  | 95.1 | 6,028 | 99.4 | 498 |  | 95.4 | 6,888 |
| Non-marital  cohabitation | - | - | 8.8 | 16 | 1.5 | 3 |  | 4.9 | 308 | 0.6 | 3 |  | 4.6 | 330 |
|  |  |  |  |  |  |  |  |  |  |  |  |  |  |  |
| *Union duration* | 1 | 24 | 13.3 | 5.2 | 13.2 | 4.6 |  | 15.2 | 5.2 | 16.9 | 4.8 |  | 15.2 | 5.2 |
| 1-10 years | - | - | 29.8 | 54 | 28.5 | 57 |  | 18.5 | 1,174 | 10.8 | 54 |  | 18.6 | 1,339 |
| 11-24 years | - | - | 70.2 | 127 | 71.5 | 143 |  | 81.5 | 5,162 | 89.2 | 447 |  | 81.4 | 5,879 |
|  |  |  |  |  |  |  |  |  |  |  |  |  |  |  |
| *Total* | *-* | *-* | *2.5* | *181* | *2.8* | *200* |  | *87.8* | *6,336* | *6.9* | *501* |  | *100* | *7,218* |

*Source*: Calculations based on GSOEP 1984-2020, N=7,218.

**Table S-1.2: Overview over the sample structure**

| *Women and their unions* | ***Number of women in the analysis sample*** | ***%*** | ***Number of observations (i.e., unions) per women in the analysis sample*** |
| --- | --- | --- | --- |
| *Women with 1^st^ unions only* | 6,885 | 97.6 | 6,885 |
| *Women with 2^nd^ unions only* | 3 | 0.1 | 3 |
| *Women with 1^st^ and 2^nd^ unions* | 165 | 2.3 | 330 |
| *N* | *7,053* | *100* | *7,218* |
| *Source*: Calculations based on GSOEP 1984-2020, N=7,218. | | | |

**Table S-1.3: Migrant (descendant)s’ country of origin with mean TFR from 1960 to 2020**

| ***Low-fertility countries*** | | | |  | ***High(er)-fertility countries*** | | | |
| --- | --- | --- | --- | --- | --- | --- | --- | --- |
| Country | ⌀TFR_(1960-2020)_ | N=567 | % |  | Country | ⌀ TFR_(1960-2020)_ | N=315 | % |
| Italy | 1.7 | 109 | 19.2 |  | Turkey | 3.7 | 183 | 58.1 |
| Poland | 1.9 | 77 | 13.6 |  | Then-Yugoslavia† | 2.2 | 22 | 7.0 |
| Greece | 1.7 | 72 | 12.7 |  | Kazakhstan | 3.0 | 16 | 5.1 |
| Spain | 1.9 | 59 | 10.4 |  | Philippines | 4.6 | 9 | 2.9 |
| Austria | 1.7 | 29 | 5.1 |  | Macedonia | 2.3 | 9 | 2.9 |
| Croatia | 1.7 | 28 | 4.9 |  | Morocco | 4.4 | 7 | 2.2 |
| Russia | 1.8 | 24 | 4.2 |  | Iran | 4.4 | 6 | 1.9 |
| Romania | 2.0 | 18 | 3.2 |  | Mexico | 4.1 | 4 | 1.3 |
| Netherlands | 1.9 | 17 | 3.0 |  | Kosovo | 3.8 | 4 | 1.3 |
| France | 2.1 | 16 | 2.8 |  | Bolivia | 4.7 | 3 | 0.8 |
| Bosnia and Herzegovina | 2.0 | 16 | 2.8 |  | Sri Lanka | 3.1 | 3 | 1.0 |
| United States | 2.1 | 13 | 2.3 |  | China | 2.9 | 3 | 1.0 |
| Great Britain | 2.0 | 11 | 1.9 |  | Vietnam | 3.8 | 3 | 1.0 |
| Switzerland | 1.7 | 11 | 1.9 |  | Chile | 2.7 | 3 | 1.0 |
| Czech Republic | 1.8 | 10 | 1.8 |  | Argentina | 2.8 | 2 | 0.6 |
| Hungary | 1.7 | 9 | 1.6 |  | Thailand | 3.0 | 2 | 0.6 |
| Slovenia | 1.7 | 9 | 1.6 |  | Tunisia | 4.0 | 2 | 0.6 |
| Serbia | 1.6 | 9 | 1.6 |  | Brazil | 3.3 | 2 | 0.6 |
| Ukraine | 1.7 | 6 | 1.1 |  | Peru | 4.2 | 2 | 0.6 |
| Canada | 1.9 | 4 | 0.7 |  | Ireland | 2.6 | 2 | 0.6 |
| Sweden | 1.9 | 3 | 0.5 |  | Egypt | 4.6 | 2 | 0.6 |
| Portugal | 2.0 | 3 | 0.5 |  | Paraguay | 4.4 | 2 | 0.6 |
| Denmark | 1.8 | 2 | 0.4 |  | Syria | 5.5 | 2 | 0.6 |
| Finland | 1.8 | 2 | 0.4 |  | India | 4.1 | 1 | 0.3 |
| Bulgaria | 1.8 | 2 | 0.4 |  | Afghanistan | 7.0 | 1 | 0.3 |
| Belgium | 1.8 | 2 | 0.4 |  | Jamaica | 3.3 | 1 | 0.3 |
| Slovakia | 2.0 | 2 | 0.4 |  | Ethiopia | 6.5 | 1 | 0.3 |
| Belarus | 1.8 | 2 | 0.4 |  | Colombia | 3.5 | 1 | 0.3 |
| Japan | 1.6 | 1 | 0.2 |  | Ghana | 5.6 | 1 | 0.3 |
| Lithuania | 1.9 | 1 | 0.2 |  | Venezuela | 3.8 | 1 | 0.3 |
|  |  |  |  |  | Nigeria | 6.3 | 1 | 0.3 |
|  |  |  |  |  | Iraq | 5.6 | 1 | 0.3 |
|  |  |  |  |  | Lebanon | 3.5 | 1 | 0.3 |
|  |  |  |  |  | Mozambique | 6.0 | 1 | 0.3 |
|  |  |  |  |  | ElSalvador | 4.1 | 1 | 0.3 |
|  |  |  |  |  | Eritrea | 5.8 | 1 | 0.3 |
|  |  |  |  |  | Costa Rica | 3.3 | 1 | 0.3 |
|  |  |  |  |  | Uzbekistan | 4.2 | 1 | 0.3 |
|  |  |  |  |  | Laos | 5.1 | 1 | 0.3 |
|  |  |  |  |  | Namibia | 5.1 | 1 | 0.3 |
|  |  |  |  |  | Dominican Republic | 4.0 | 1 | 0.3 |
|  |  |  |  |  | Uruguay | 2.4 | 1 | 0.3 |
|  |  |  |  |  | Cameroon | 5.8 | 1 | 0.3 |
|  |  |  |  |  | Georgia | 2.2 | 1 | 0.3 |
|  |  |  |  |  | Armenia | 2.5 | 1 | 0.3 |
|  |  |  |  |  | Liberia | 6.0 | 1 | 0.3 |
|  |  |  |  |  | Palestine | 2.5 | 1 | 0.3 |

*Source*: Calculations based on The World Bank (Data) 2023, “Fertility rate, total (births per woman)” available online at: https://data.worldbank.org/indicator/SP.DYN.TFRT.IN, N=882.

*Notes:* † Average of the mean TFRs of Croatia, Bosnia and Herzegovina, Macedonia, Slovenia, Kosovo, Montenegro (⌀ TFR=2.2), and Serbia.
